# Performance of Deep Learning Reconstruction for Detection of Early Ischemic Changes in NCCT: Comparison with ASIR-V in Acute Stroke

**DOI:** 10.1007/s00062-026-01645-5

**Published:** 2026-04-09

**Authors:** Michal Pula, Adrian Korbecki, Krzysztof Winiarczyk, Arkadiusz Kacała, Kamil Litwinowicz, Michal Sobanski, Julita Nadwodna, Agata Zdanowicz-Ratajczyk, Maciej Guziński

**Affiliations:** 1 Department of General Radiology, Interventional Radiology and Neuroradiology, Wroclaw Medical University Hospital, Wrocław, Poland; 2 Emergency Medicine Center, Lower Silesian Specialist Hospital T. Marciniak, Wrocław, Poland; 3 Department of General Radiology, Interventional Radiology and Neuroradiology, Wroclaw Medical University, Wrocław, Poland; 4 Hetalox sp. z o.o., Wrocław, Poland

**Keywords:** DLIR, Stroke, NCCT, ASPECT, Deep Learning

## Abstract

**Purpose:**

Evaluation of the impact of Deep Learning Image Reconstruction (DLIR) compared to Adaptive Statistical Iterative Reconstruction-Veo (ASIR-V) on image quality and early ischemic changes detection on non-contrast computed tomography (NCCT) in stroke suspected patients. A secondary objective was to determine the potential influence of reconstruction algorithm on ASPECT scoring relative to automated e-ASPECT score.

**Methods:**

Consecutive patients undergoing NCCT within 6 hours of symptom onset were retrospectively included. Images were reconstructed using ASIR-V and high-strength DLIR. Four readers with varying experience independently assessed subjective image quality, gray–white matter differentiation, diagnostic confidence, presence of ischemic lesions and ASPECTS scoring. Diagnostic performance (accuracy, sensitivity, specificity) was calculated using e-ASPECTS as reference. Evaluation time was recorded.

**Results:**

DLIR significantly improved subjective image quality and gray–white matter contrast compared with ASIR-V (odds ratios 2.96–3.96; p 0.001). Diagnostic performance for detecting early ischemic changes showed no significant difference, with similar accuracy, sensitivity and specificity. Evaluation time did not differ. A trend toward higher specificity and reduced bias for ASPECTS ≥6 was observed with DLIR, but mixed-model analysis did not confirm statistical significance.

**Conclusion:**

DLIR improves subjective image quality in acute stroke NCCT but does not significantly improve detection accuracy or ASPECTS scoring compared with ASIR-V. A tendency toward improved specificity was observed; further studies with larger cohorts are needed.

## Introduction

Cerebral stroke is the second leading cause of death and the third leading cause of disability worldwide with an overwhelming burden on both society and individual patients [1]. Among other types of strokes, the primary cause of brain ischemia is the large vessel occlusion (LVO). It is an important subset of ischemic stroke (IS) as it is associated with more than double the risk of death and permanent disability as compared to non-LVO IS [2].

Advances in medical technology have introduced a wide range of diagnostic tests and treatment options for managing acute stroke. Radiological imaging plays a critical role in this process, and current guidelines strongly recommend neuroimaging for all patients with suspected acute stroke [3]. A multitude of diagnostic tests enables tailoring the diagnostic process to each patient’s specific needs. For instance, magnetic resonance imaging techniques are valuable for evaluating therapeutic options, particularly in cases where the exact onset of symptoms is unknown or a suspected lesion is located within the brainstem [4]. Similarly, vessel imaging techniques such as digital subtraction angiography (DSA) or CT angiography are essential for assessing patients who may be candidates for endovascular treatment [5]. Nevertheless, the primary imaging modality for suspected stroke cases remains non-contrast enhanced CT (NCCT), owing to its broad availability, cost-effectiveness, and, most importantly, its relatively short examination time and reduced susceptibility to patient-related disturbances—crucial for rapid treatment introduction [6].

The diagnostic accuracy of CT imaging is highly dependent on image quality parameters, including noise, contrast, resolution, and artifacts [7]. Since its introduction, continuous efforts have been made to improve CT image quality. Conversely, an important factor influencing image quality in CT examinations is the radiation dose. While it is important to minimize the absorbed dose to patients, given that examinations using ionizing radiation contribute significantly to the cumulative absorbed dose in society, this reduction requires compromises in image quality [8]. Therefore, it is necessary to provide image quality that is diagnostically appropriate to the health issue presented by the patient rather than the perfect image that requires a higher radiation dose.

One of the features contributing to radiation dose reduction while preserving the image quality at the diagnostic level is the reconstruction protocols. Older algorithms, such as simple back projection and filtered back projection, are being replaced with their more advanced successors, such as Adaptive Statistical Iterative Reconstruction Veo (ASIR-V) and the novel algorithm utilizing deep learning networks—Deep Learning Image Reconstruction (DLIR). Current research indicates that DLIR offers superior image quality compared to ASIR‑V [9]. However, evidence regarding the impact of reconstruction type on sensitivity and specificity is limited in available studies. Most papers focused solely on the comparison of objective parameters, lacking the assessment of the impact of the reconstruction type on radiologist evaluation of the examination.

The growing diagnostic abilities of all the imaging modalities have led to a significant increase in the number of examinations performed each year. Unfortunately, the number of radiology specialists is insufficient to meet the ever-increasing demand for interpretation of the performed studies [10]. Therefore, it is necessary to seek new solutions allowing for acceleration of the workflow without compromising the quality of prepared reports. One of the possible solutions to this problem might be the optimization of image quality allowing for quicker assessment of the examination. Another potential factor that shortens the time required for examination evaluation and allows for preselection of studies requiring the most urgent evaluation is artificial intelligence (AI) tools that support physicians’ decision-making. Due to the rapid development of AI, numerous supporting tools, which demonstrated significant positive impact on the speed of imaging diagnostics and treatment implementation, are now available [11]. One such tool is the Brainomix 360 Stroke platform. This tool uses AI algorithms to evaluate NCCT examinations in real time and has the ability to identify ischemic lesions, bleeding, and areas susceptible to reperfusion if treatment is implemented quickly.

This study was conducted to compare time efficacy, image quality, and the examination ability to evaluate stroke lesions in NCCT scans performed with DLIR compared to ASIR‑V in patients with suspected acute ischemic stroke (AIS) under Emergency Department (ED) conditions. Furthermore, the study compared the results provided by radiologists using both approaches with the scores assigned by the Brainomix software.

## Materials and Methods

In this study, a retrospective analysis of NCCT examinations in 50 patients (age median 72.5 years; 32 female and 18 male) who underwent imaging for suspected ischemic stroke were assessed. All the examinations were performed in the emergency department between January 2023 and December 2024. Demographic characteristics of the study population are presented in Table 1.

**Table 1:** Demographic characteristic of study population

Gender	*n* (%)	Age Median (IQR)	ASPECT Median (IQR)	AIS Present *n* (%)
Male	18 (36%)	71 (67.25–75.5)	8 (6.25–9)	16 (88.9%)
Female	32 (64%)	73 (67.75–85.75)	9 (7–9.25)	24 (75%)
Total	50 (100%)	72.5 (67.25–83)	8 (7–9)	40 (80%)

Patient selection was based on the results generated by the Brainomix software, which provided an automated electronic Alberta Stroke Program Early CT Score (e-ASPECTS), in concordance with radiology reports retrieved from the hospital’s internal documentation system. Studies were included only when the lesion identified by Brainomix was confirmed by a radiologist to represent an acute ischemic lesion. Cases in which Brainomix incorrectly classified prior ischemic lesions, tumors, or areas of edema as acute ischemia were excluded from the analysis.

**Fig. 1:**
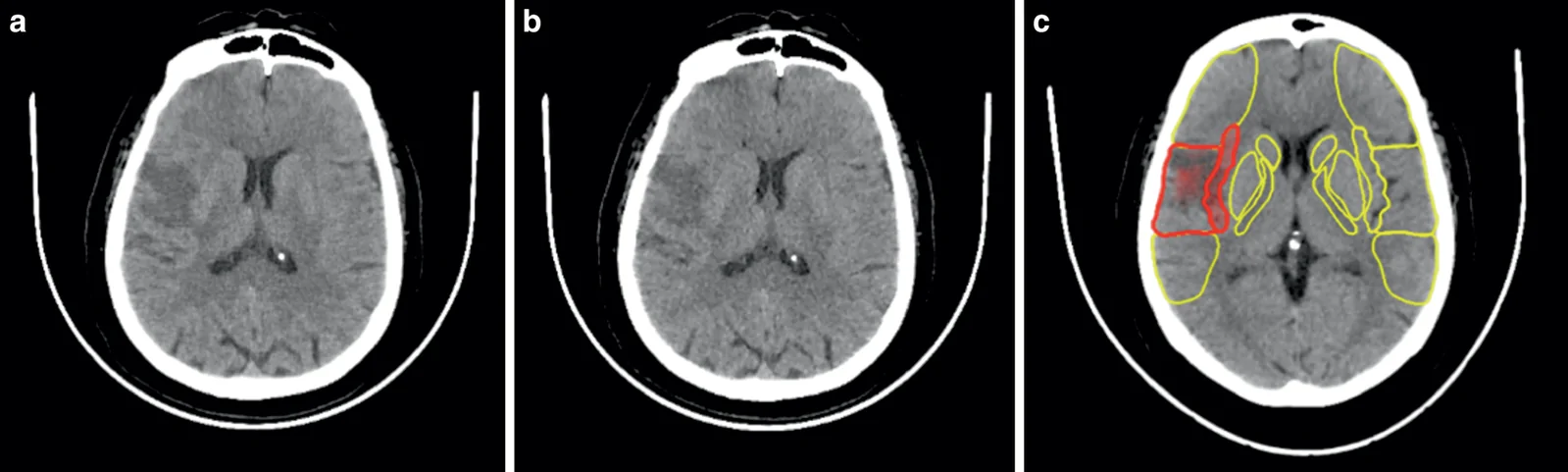
NCCT examination reconstructed with DLIR (**a**), ASIR-V (**b**), and with Brainomix augmentation (**c**)

The final cohort comprised 40 NCCT examinations with a Brainomix e‑ASPECTS score < 10 and 10 examinations with a score of 10. The first group was considered the study group with varying severity of ischemic lesions present in the scans, while the second was considered the control group with the scans free of any pathology reported by both the Brainomix and the reviewing radiologist. The distribution of e‑ASPECTS scores within the study population is presented in Table 2. The stroke suspected patient examination with its Brainomix augmentation was presented in Fig. 1.

**Table 2:** Reference e‑ASPECT score distribution

ASPECT Score	*n* (%)
2	2 (4%)
3	1 (2%)
4	4 (8%)
5	2 (4%)
6	2 (4%)
7	8 (16%)
8	8 (16%)
9	13 (26%)
10	10 (20%)

All of the examinations were performed using a 256-row Revolution Apex CT scanner (GE Healthcare) with axial acquisition. Patients were positioned supine for the examination. Tube voltage was set to 120 kVp, tube current to 320 mA, beam collimation to 80 mm, slice thickness to 0.625 mm, and a scan range extending from the foramen magnum to the vertex for all of the examination. The gantry rotation time was 1 s, and automatic exposure control (AEC) was enabled.

Image reconstruction was performed using deep learning image reconstruction (DLIR) with TrueFidelity (GE Healthcare), a deep neural network engine trained on high-quality filtered back projection (FBP) images. The highest strength setting (DLIR-H) was applied. For research purposes, images were also reconstructed using ASIR‑V with a hybrid approach of 50% ASIR‑V and 50% FBP. The Brainomix e‑ASPECT reports were generated from the ASIR‑V reconstructed NCCT sequences, uploaded into the e‑stroke platform by the performing technician, in accordance with the center-own protocol.

The preselected scans were then uploaded to a single workstation. The results were randomized for patients’ personal data, and reconstruction type information. The initial window width and level of all images were 80 and 40 HU, respectively, and the readers were able to adjust the window width and window level to the position considered appropriate. Each examination was randomly assigned a number from 1 to 100. All CT scans were evaluated independently by four readers using a structured, web-based secured questionnaire specifically designed for this study. The readers had 5, 6, 8 and 10 years of experience in neuroradiology, respectively. Every reader was assessing the examinations individually.

The questionnaire consisted of eight questions addressing both technical and diagnostic aspects of image interpretation. First, readers rated the overall image quality using a 5-point Likert scale (1 = non-diagnostic, 5 = excellent). Then they binary assessed the presence or absence of AIS. In cases where AIS was identified, readers indicated the lesion’s anatomical location using a multiple-choice list of brain regions (frontal, temporal, parietal, occipital lobes, basal ganglia, left/right distribution).

Next, readers provided a subjective ASPECTS score, supported by a standardized reference diagram. The remaining questions captured readers’ impressions of the image’s diagnostic ability for detecting acute ischemic lesions and its ability to visualize corticomedullary differentiation and delineate the internal capsule, each scored on a 5-point Likert scale (1 = non-diagnostic, 5 = excellent).

For each case, the time taken to complete the assessment was automatically recorded by the online platform. All readers were blinded to each other’s responses. Responses were exported from the platform into a structured spreadsheet format for subsequent statistical analysis.

All analyses were performed in R (v4.4.2). Between-method comparisons for quality metrics and ASPECT bias were made using cumulative link mixed models (CLMMs)with Method (ASIR vs. DLIR) as a fixed effect and random intercepts for Patient and Reader, yielding odds ratios (OR) and 95% confidence intervals (CI). Reader-specific quality ratings were compared via Wilcoxon tests. Reading Times were compared using paired t‑tests across matched ASIR/DLIR readings and further evaluated with linear mixed-effects models (LMMs; fixed effect: Method; random intercepts: Patient, Reader). Detection of acute ischemic lesions (AIL) and ASPECT ≥ 6 was assessed by sensitivity, specificity, PPV, NPV, and overall accuracy; hierarchical logistic regression (GLMM) with Method as fixed effect and Patient/Reader random effects provided OR (95% CI) for correct detection.

## Results

### Subjective Image Quality

Across all readers, DLIR reconstructions achieved consistently higher subjective image quality ratings compared with ASIR. Median scores in Likert scale for questions 1, 5, 6 and 7 (regarding overall image quality, diagnostic ability, corticomedullary differentiation, and internal capsule differentiation respectively) were all significantly higher for DLIR (*p* < 0.001 for all metrics). Cumulative link mixed model (CLMM) analysis confirmed that DLIR significantly increased the odds of achieving higher quality ratings, with odds ratios (OR) ranging from 2.96 to 3.96 (Table 3).

**Table 3:** Image Quality Summary Statistics by Method. Cumulative link mixed models with Patient and Reader random effects.

Quality Metric	ASIR Median (IQR)	DLIR Median (IQR)	OR (95% CI)
Corticomedullary Differentiation	3 (2–4)	4 (3–4)	3.82 (2.56–5.70)***
Diagnostic Ability	3 (3–4)	4 (3–4)	3.48 (2.31–5.25)***
Internal Capsule Differentiation	3 (2–4)	4 (3–4)	3.96 (2.66–5.88)***
Overall Image Quality	3 (2–4)	4 (3–4)	2.96 (1.98–4.41)***

Values shown as median (Q1–Q3). OR > 1 indicates higher odds of better ratings with DLIR. Significance: *** *p* < 0.001, ** *p* < 0.01, * *p* < 0.05.

### Inter-Rater Reliability

**Fig. 2:**
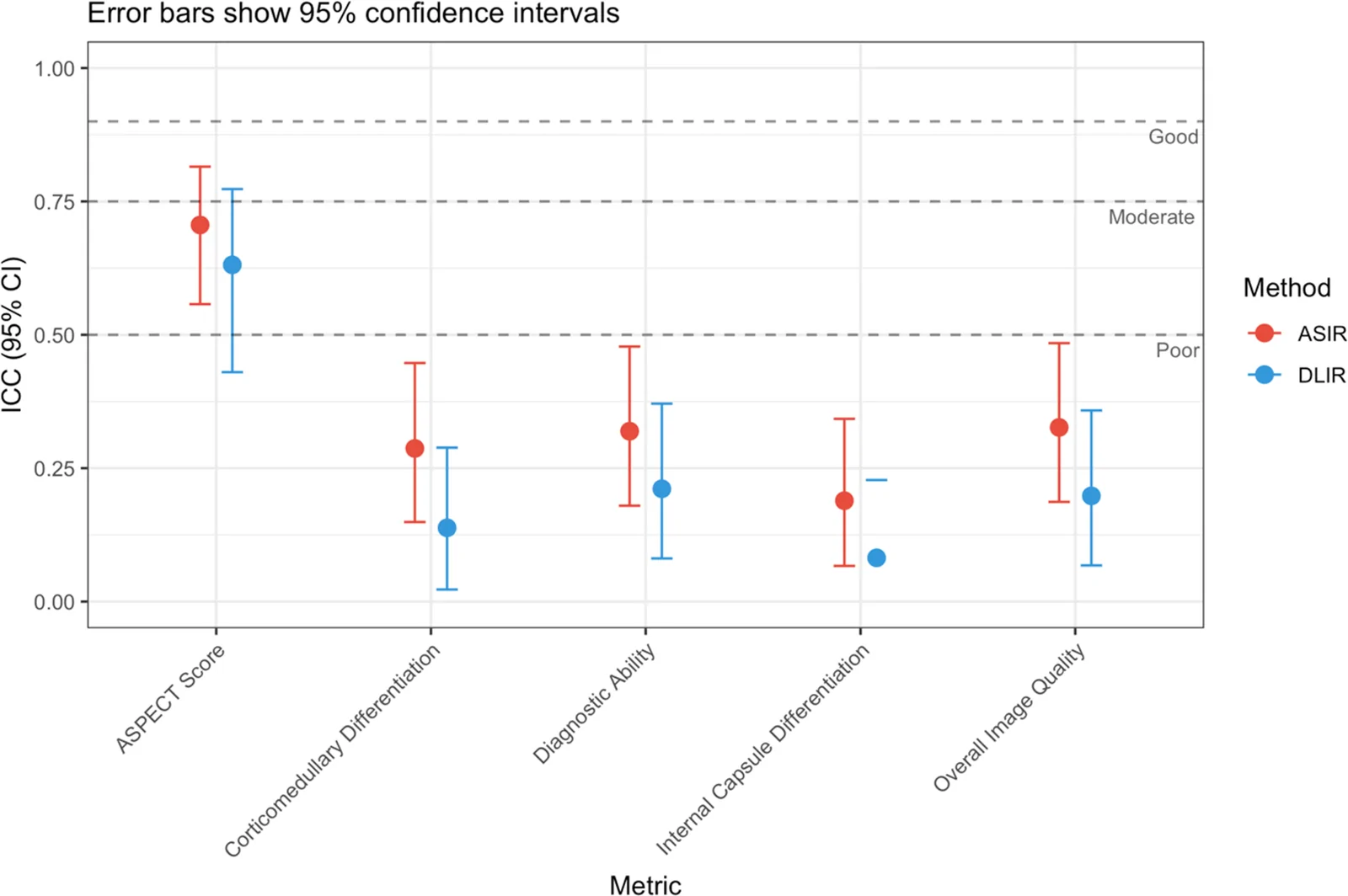
Inter-Reader Reliability (ICC) comparison for ASIR vs DLIR

**Fig. 3:**
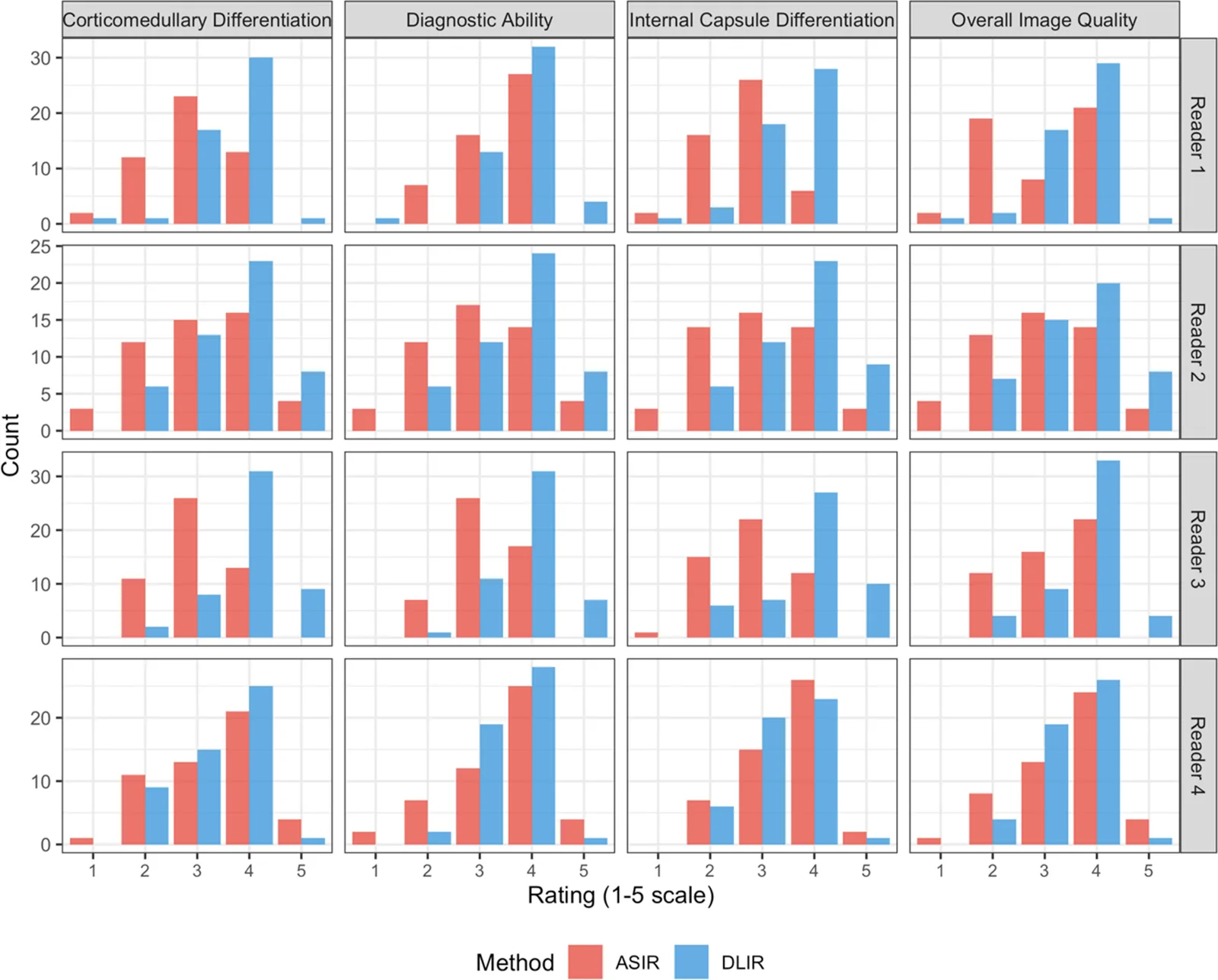
Quality ratings likert-scale score distributions by reader’s years’ experience in neuroradiology (Reader 1–10 years, Reader 2–6 years, Reader 3–8 years, Reader 4–5 years)

Inter-rater agreement for image quality ratings was poor across both methods (ICC = 0.08–0.33), with even lower values for DLIR examinations. However, there were no statistically significant differences in ICC across both methods. Reliability for ASPECT scoring was moderate for both ASIR (ICC = 0.71) and DLIR (ICC = 0.63) (Table 4). No metric achieved good or excellent reliability (Fig. 2 and 3).

**Table 4:** Inter-Rater Reliability (ICC) for Image Quality Metrics

Method	Quality Metric	ICC (95% CI)	*p*-value	Reliability
ASIR	Overall Image Quality	0.326 (0.187–0.484)	< 0.001	Poor
ASIR	Diagnostic Ability	0.319 (0.180–0.478)	< 0.001	Poor
ASIR	Corticomedullary Differentiation	0.287 (0.149–0.447)	< 0.001	Poor
ASIR	Internal Capsule Differentiation	0.189 (0.067–0.343)	< 0.001	Poor
DLIR	Overall Image Quality	0.198 (0.068–0.358)	< 0.001	Poor
DLIR	Diagnostic Ability	0.211 (0.081–0.371)	< 0.001	Poor
DLIR	Corticomedullary Differentiation	0.138 (0.023–0.289)	0.008	Poor
DLIR	Internal Capsule Differentiation	0.082 (−0.027–0.228)	0.076	Poor

### Reading Time

Average interpretation times were comparable between ASIR and DLIR (139 ± 80.8 s vs. 137 ± 62.8 s, respectively). Linear mixed-effects modelling demonstrated no significant difference between reconstruction methods (β = −2 s, 95% CI −15.4 to 11.4; *p* = 0.77). Reading times also presented no significant reader-specific effect (Table 5).

**Table 5:** Reading Time Summary Statistics Reader-by-Reader for each Methode (seconds).

Reader	ASIR (mean ± SD)	DLIR (mean ± SD)
1	147.18 ± 38.79	152.04 ± 55.71
2	157.14 ± 99.14	153.38 ± 49.30
3	118.36 ± 109.38	104.02 ± 80.07
4	133.44 ± 48.33	138.68 ± 49.68
Overall	*139* *±* *80.8*	*137* *±* *62.8*

### Detection of Acute Ischemic Lesions (AIL)

Overall, no statistically significant difference in diagnostic performance for detecting AIL was recorded with DLIR over ASIR‑V, in terms of accuracy and sensitivity (80% vs 76% and 77.5% vs 70.6%, respectively). However, ASIR‑V examinations allowed for higher specificity 90% vs 97.5% for DLIR and ASIR‑V (Table 6). Positive and negative predictive values were 96.9% and 50.0% for DLIR, and 99.1% and 45.3% for ASIR. Hierarchical logistic regression showed no statistically significant difference in detection accuracy between methods (OR = 1.58, 95% CI 0.81–3.09; *p* = 0.18).

**Table 6:** Reader-Wise AIL detection performance

	Accuracy (%)		Sensitivity (%)		Specificity (%)	
Reader	ASIR	DLIR	ASIR	DLIR	ASIR	DLIR
1	84	92	82.5	92.5	90	90
2	84	78	80.0	80.0	100	70
3	72	84	65.0	80.0	100	100
4	64	66	55.0	57.5	100	100

### Detection of ASPECT ≥ 6

Performance in identifying cases with ASPECT ≥ 6 was high for both reconstruction methods.

Although both recontruction methods achived smiliar overall accuracy (86.5% vs. 84.0%), and identical sensitivity (92.1% for both), DLIR reconstructions provided improved specificity (61.1% vs. 47.2%) (Table 7). Logistic regression again showed no significant difference between methods (OR = 1.42, 95% CI 0.68–2.98; *p* = 0.35).

**Table 7:** Mean and Reader-Wise ASPECT ≥ 6 detection performance.

Reader/Method	Accuracy (%) ASIR‑V	Accuracy (%) DLIR	Sensitivity (%) ASIR‑V	Sensitivity (%) DLIR	Specificity (%) ASIR‑V	Specificity (%) DLIR
1	80	88	85.4	90.2	55.6	77.8
2	86	86	92.7	87.8	55.6	77.8
3	84	88	90.2	90.2	55.6	77.8
4	86	84	100.0	100.0	22.2	11.1
Overall (Mean)	*84.0*	*86.5*	*92.1*	*92.1*	*47.2*	*61.1*

### ASPECT Scoring Accuracy

**Fig. 4:**
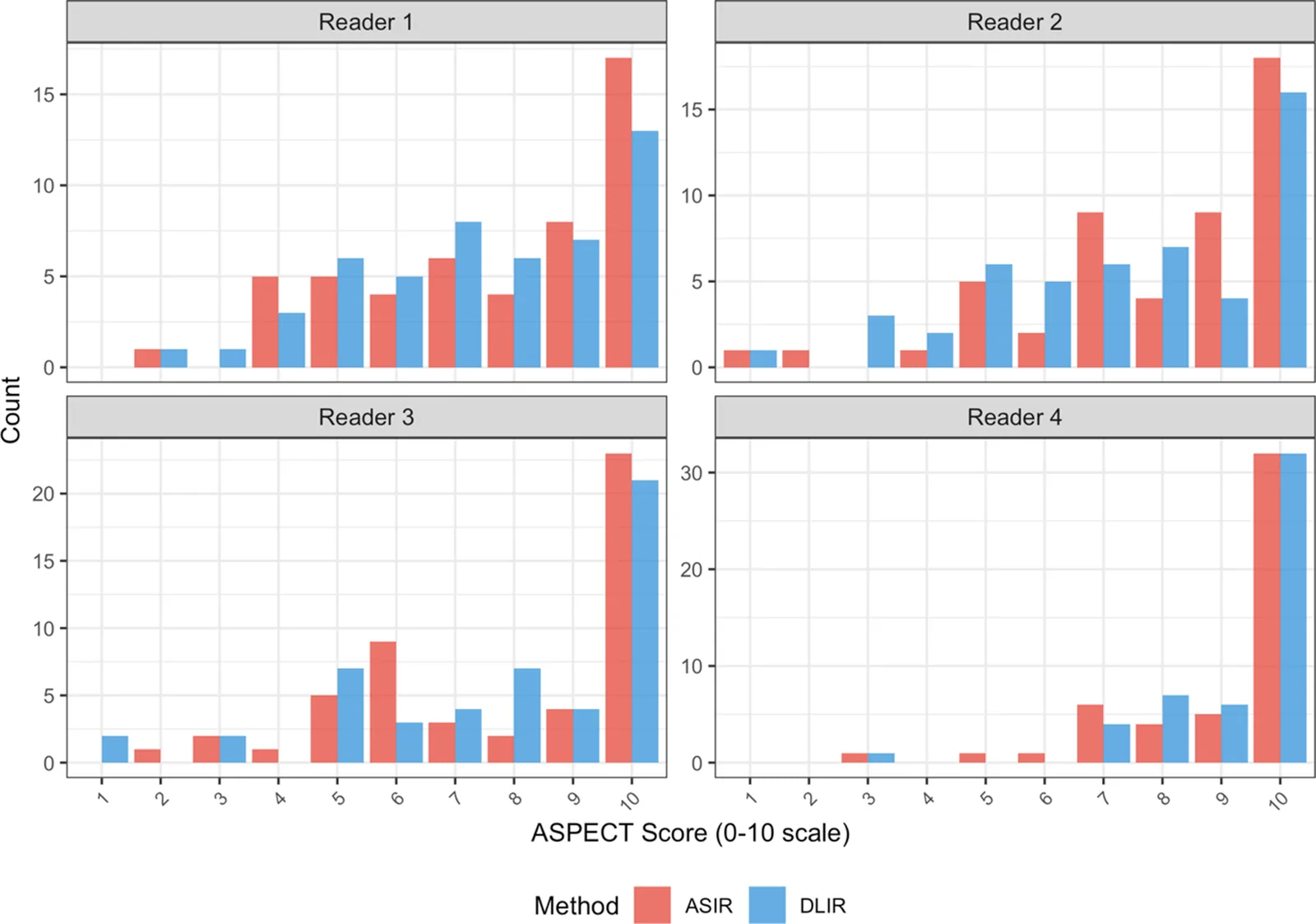
ASPECT score distribution by Reader

Median subjective ASPECT scores were identical between ASIR and DLIR (median = 9, IQR 7–10). Mean bias relative to reference was lower for DLIR (0.35 ± 1.78) compared with ASIR (0.53 ± 1.75), and both methods produced nearly identical mean absolute error (1.29 vs. 1.28). Reader-level analysis demonstrated similar trends for all readers except one, who showed a small positive bias for both methods (Table 8, and Fig. 4). CLMM analysis revealed no statistically significant difference in scoring bias between reconstruction methods (OR = 0.74, 95% CI 0.52–1.07; *p* = 0.11).

**Table 8:** Reader-Wise ASPECT scoring performance in DLIR and ASIR examinations.

Reader	Mean Bias (ASIR-V)	Mean Bias (DLIR)	Mean Abs Error (ASIR-V)	Mean Abs Error (DLIR)	RMSE (ASIR-V)	RMSE (DLIR)	Within 1 Point (ASIR-V)	Within 1 Point (DLIR)
1	0.10	−0.16	1.18	1.12	1.57	1.55	62	66
2	0.36	−0.20	1.24	1.24	1.83	1.62	64	72
3	0.24	0.18	1.16	1.10	1.52	1.52	68	76
4	1.42	1.58	1.54	1.70	2.27	2.40	62	54

### AI Enhancement Recognition

Readers correctly identified DLIR images as AI-enhanced in 77% of cases and ASIR as non-AI in 67% of cases, yielding an overall classification accuracy of 72%. Individual reader accuracy ranged from 57% to 81%, without a consistent trend between experience and detection accuracy (Table 9).

**Table 9:** AI enhancement recognition—binary classification metrics

Performance Metric	Value (%)	Interpretation
Overall Accuracy	72	Correct classifications across all images
Sensitivity (AI Detection)	77	Correctly identified DLIR as AI-enhanced
Specificity (Non-AI Detection)	67	Correctly identified ASIR‑V as non-AI-enhanced
PPV	70	When readers said ’AI’, % that were correct
NPV	74.4	When readers said ’No AI’, % that were correct

## Discussion

In this study, a comprehensive subjective assessment of NCCT images reconstructed using DLIR and ASIR‑V in emergency department patients with suspected ischemic stroke was performed. The utilization of a complex questionnaire, including four different questions related to image quality in terms of differentiating recent ischemic lesions, aimed to provide a comprehensive assessment of the potential of new machine learning-based reconstruction methods (DLIR) to improve diagnostic performance in patients with suspected ischemic stroke. In all four questions (overall image quality, diagnostic ability, corticomedullary differentiation, and internal capsule differentiation), images reconstructed using DLIR achieved statistically significantly higher scores compared to ASIR‑V images. Our results are consistent with the observations by Alagic et al. and Kim et al. [9, 12]. However, in our study, the patient group was specifically selected for patients with suspected ischemic lesions in the brain, in contrast to the trauma and lesion-free patients in the aforementioned studies, respectively. Furthermore, in our study, in order to reduce the risk of bias arising from overly uniform reader group, the assessment was performed by four radiologists with varying degrees of expertise. The radiologists in our study were blinded to both the image reconstruction model and the results of the other readers. The subjective ratings of image quality for head CT reconstructions exhibited poor inter-reader reliability, indicating considerable variability between the four neuroradiologists in how they assessed the scans. The reconstruction algorithms such as DLIR and ASIR‑V modify image features such as noise texture and edge to a different extent, and these changes may be interpreted differently by the readers. Moreover, the nature of Likert-type scales (used for image quality) introduces interpretive variability: one reader’s “4 = good” may correspond to another’s “3 = acceptable”. This result distinguishes our study from the results obtained by Kim et al., who obtained moderate to good inter-reader agreement. This may suggest that subjective quality assessments may be significantly influenced by varying levels of experience among individual readers (similar in the Kim et al. study, varying in our study). Although direct comparison between ICC and weighted kappa is not statistically valid, our lower agreement values suggest greater variability among readers in subjective image assessment than previously reported. Although DLIR reconstructions in this study exhibited higher median ratings, the divergent subjective scores across readers suggest that algorithm advantages may not uniformly translate into reader perception. Consequently, adoption of a new reconstruction method (e.g., DLIR) cannot rely solely on subjective image quality assessments.

Another feature assessed in this study was the time required to evaluate the examination. For each of the assessed examinations, the reader had to complete the same form, and the timer started when the examination was opened at the station. Our study did not demonstrate a significant difference in the time required to evaluate examinations reconstructed using DLIR or ASIR (139 ± 80.8 s vs. 137 ± 62.8 s, *p* = 0.77). However, it should be noted that our results were obtained using only one type of examination in a selected study group. A comprehensive assessment requires expanding the study to include more diverse study groups and other types of CT examinations.

In the present study, readers demonstrated moderate ability to discriminate between AI-enhanced (DLIR) and conventionally reconstructed (ASIR) images, achieving an overall classification accuracy of 72%. A sensitivity of 77% indicates that DLIR images were correctly identified as AI-enhanced in most cases, whereas a specificity of 67% indicates that ASIR images were less consistently identified as non-AI. The positive and negative predictive values (70% and 74.4%, respectively) suggest balanced performance, with no bias toward either reconstruction type. Individual reader accuracy ranged from 57% to 81%, and no clear correlation between reader experience and accuracy was observed. These findings suggest that although AI-based reconstructions exhibit discernible differences recognizable by most observers, the variability among readers highlights that visual features introduced by DLIR may not be consistently distinguishable, particularly in routine clinical settings.

In our cohort of NCCT examinations, DLIR reconstruction yielded a modest improvement in overall ischemic lesion detection (accuracy 80% vs 76%) and sensitivity (77.5% vs 70.6%) compared with ASIR, while ASIR retained higher specificity (97.5% vs 90.0%). The effect of DLIR, however, was reader-dependent: two readers demonstrated gains in sensitivity and accuracy, whereas others showed minimal change or lack of impact of the reconstruction algorithm on lesion assessment. The hierarchical logistic model produced a non-significant odds ratio favouring DLIR (OR 1.58, 95% CI 0.81–3.09; *p* = 0.18), indicating that the observed improvement did not reach statistical significance after accounting for reader- and patient-level clustering. These findings suggest that DLIR may enhance lesion conspicuity and reduce false negatives in some hands, but benefits are heterogeneous and must be validated in larger, multicentre cohorts. Importantly, high PPVs and relatively low NPVs indicate that positive reads were reliable, while negative reads were less reassuring, a pattern influenced by lesion prevalence and case selection. Future work should focus on whether DLIR-related improvements in sensitivity translate into clinically meaningful changes in acute stroke triage and outcomes, and whether targeted reader training can reduce between-reader variability.

Our findings are in line with those of Sun et al., who reported higher lesion detection rates with DLIR compared to ASIR‑V, although their analysis involved different slice thicknesses in head CT [13]. Similarly, Yang et al. demonstrated superior diagnostic performance of DLIR over ASIR‑V in contrast-enhanced abdominal CT, with the most favourable results achieved using DLIR of medium-strength (DLIR-M) [14]. Consistent with this, Bie et al. observed that, despite DLIR of high-strength (DLIR-H) providing greater noise reduction than DLIR‑M and DLIR of low-strength (DLIR-L) settings, the contrast-to-noise ratio—critical for the visibility of small focal lesions—was higher with the latter two [15]. In our study, all images were reconstructed using DLIR‑H. It is therefore conceivable that employing DLIR‑M could further enhance focal lesion detection, although this hypothesis requires additional investigation.

In our cohort, DLIR reconstruction produced a slight but consistent reduction in positive scoring bias compared with ASIR‑V (mean bias 0.35 vs 0.53) and increased the proportion of reads within ±1 ASPECT point (67% vs 64%), while mean absolute error and RMSE remained essentially unchanged. Reader-level effects were heterogeneous: three readers showed improved proximity to reference scores with DLIR, whereas one reader maintained a marked positive bias regardless of reconstruction method. A cumulative link mixed model yielded a non-significant trend toward reduced scoring bias with DLIR (OR 0.74, 95% CI 0.52–1.07; *p* = 0.11). The reduction in positive bias is important because over-scoring ASPECTS can inappropriately broaden eligibility for reperfusion therapies; DLIR therefore has the potential to modestly reduce such misclassification. However, the scale of improvement and enduring inter-reader variability indicate that reconstruction improvements alone are unlikely to eliminate scoring inconsistency. DLIR reconstruction was associated with a slight increase in the overall accuracy of ASPECT ≥ 6 detection (86.5% vs. 84.0% for ASIR-V) and a marked improvement in specificity (61.1% vs. 47.2%), while sensitivity remained unchanged (92.1% for both methods). Clinically, this finding is relevant because DLIR did not compromise the ability to identify patients eligible for reperfusion therapy, while simultaneously reducing the number of false-positive classifications potentially limiting inappropriate qualification for invasive procedures. The benefits of DLIR, however, varied between readers: two radiologists demonstrated a clear improvement, whereas one maintained a high sensitivity reading strategy at the expense of a substantial number of false positives. After adjusting for random effects of patient and reader in a hierarchical model, the advantage of DLIR did not reach statistical significance (OR 1.42, *p* = 0.35). These results highlight the necessity for centre-own validation and reader training before clinical implementation of DLIR in AIS imaging.

Our study had several limitations that should be considered when interpreting the obtained results. First, it was conducted at a single centre on a relatively small group of patients, which limits the generalizability of the results and their statistical validity. Moreover, patient inclusion in the study was based on the consistency of radiological descriptions and e‑ASPECTS scores generated by Brainomix software, therefore the study cohort may be susceptible to selection bias related to subjective elements of radiological assessment. Second, the study group consisted mostly of ASPECT > 6 examinations (Table 2), this resulted from the fact that all examinations were performed in the acute phase of stroke, within 6 h from symptom onset, during which most patients still present only limited hypodense changes. Consequently, the small representation of ASPECTS < 6 cases was expected. However, it restricts the ability to evaluate algorithm performance in more severe stroke phenotypes. Third, all examinations were performed using a single scanner, with the same image acquisition settings, with only one DLIR reconstruction strength—DLIR‑H. Our results may not be applicable to examinations performed on scanners from other manufacturers, and using different image acquisition or reconstruction parameters. Furthermore, the heterogeneous experience of the reader group, although deliberately selected to obtain more universal results, resulted in poor inter-reader agreement in subjective assessment of image quality. Moreover, our standard for referencing the results was e‑ASPECT and radiological descriptions, which may introduce classification bias. Utilization of follow-up imaging confirmation or pathological examinations as the standard could reduce this error. The readers were formally blinded to the reconstruction protocol; however, they were able to distinguish between the compared techniques with an accuracy exceeding 72%, which may have influenced their subjective assessments. Finally, although we assessed reading time and diagnostic accuracy, clinical outcome measures (e.g. treatment decisions or patient prognosis) were not included, hence, the practical impact of DLIR on real-world stroke management remains to be established.

## Conclusion

Deep learning image reconstruction significantly improved the image quality for NCCT compared to ASIR‑V. However, this improvement did not translate into a statistically significant increase in diagnostic accuracy or ASPECT scoring performance for ischemic lesion detection.

## Data Availability

No datasets were generated or analysed during the current study.
